# Co-designing inflammatory bowel disease (Ibd) services in Scotland: findings from a nationwide survey

**DOI:** 10.1186/s12913-016-1490-7

**Published:** 2016-07-08

**Authors:** Mariyana Schoultz, Leah Macaden, Angus J. M. Watson

**Affiliations:** School of Health Sciences, University of Stirling, Highland Campus, Inverness, UK

**Keywords:** Inflammatory bowel disease, Co-designing, Qualitative study, Patient survey, Crohn’s disease, Ulcerative colitis

## Abstract

**Background:**

The Scottish Government’s ambition is to ensure that health services are co-designed with the communities they serve. Crohn’s and Colitis UK and the Scottish Government acknowledged the need to review and update the current IBD care model. An online survey was conducted asking IBD patients about their experiences of the NHS care they receive. This survey was the first step of co-designing and developing a national strategy for IBD service improvement in Scotland.

Aim: To explore IBD patients’ experiences of current services and make recommendations for future service development.

**Methods:**

This study was part of a wider cross-sectional on-line survey. Participants were patients with IBD across Scotland. 777 people with IBD took part in the survey. Thematic analysis of all data was conducted independently by two researchers.

**Results:**

Three key themes emerged:

Quality of life: Participants highlighted the impact the disease has on quality of life and the desperate need for IBD services to address this more holistically.

IBD clinicians and access: Participants recognised the need for more IBD nurses and gastroenterologists along with better access to them. Those with a named IBD nurse reported to be more satisfied with their care.

An explicit IBD care pathway: Patients with IBD identified the need of making the IBD care pathway more explicit to service users.

**Conclusions:**

Participants expressed the need for a more holistic approach to their IBD care. This includes integrating psychological, counselling and dietetic services into IBD care with better access to IBD clinicians and a more explicit IBD care pathway.

**Electronic supplementary material:**

The online version of this article (doi:10.1186/s12913-016-1490-7) contains supplementary material, which is available to authorized users.

## Background

### Introduction

Crohn’s and Colitis UK and the Scottish Government’s ambition is to ensure that health services are co-designed with the communities they serve. Co-design is becoming an increasingly popular process in many organizations [1]. However, it is not always very clear how co-design can contribute to a service improvement project. The aim of this survey was to explore IBD patients’ perspectives and experiences of current services central to the co-designing process to direct the service improvement [2–5].

However, over the last decade, research on patients’ perspectives on care in the UK and US has produced information about variations in experiences of services and standards of care across geographical areas and hospitals [6–8]. Similar findings were recorded with the first national UK audit on IBD services and care in 2006, discovering substantial local variation in the provision, organization and clinical quality of services with aspects of care not meeting clinical guidelines [9]. The findings prompted a UK-wide strategy to improve services and care for patients who have Ulcerative Colitis or Crohn’s Disease. The strategy produced collaboration between patients and professionals, defining the minimum standards for patient-centered and high quality IBD services in 2009. The update of this strategy in 2013 suggests that significant improvements were made in IBD care and service delivery; however it also identified deficits in certain aspects of provision across the board [10]. Acknowledging this report, Crohn’s and Colitis UK and the Scottish Government recognised the need to review and update the current IBD care model in Scotland to a model that fully reflects the range of needs of patients with IBD.

### Inflammatory bowel disease

IBD is a group of chronic gastrointestinal diseases with relapsing nature and unpredictable disease course. The disease affects around 28 million people worldwide and around 250,000 patients in the UK [11, 12]. The incidence of IBD in the UK is on the increase, with up to a 76 % increase in Scotland since the mid-1990s, making Scotland the highest UK region with an incidence of 0.65 per 10.000 per year ([13, 14].

Although IBD is principally thought to be a disease of the intestinal system, a purely gastrointestinal (GI) centric view of the illness is no longer sufficient [15]. The complexity of IBD is becoming well recognized. In addition to the multiple challenges in the disease management caused by the nature of the disease (incurable, unpredictable symptoms and severity), medication side effects and surgery; the psychological stress and burden from the disease is an important part of the disease experience for patients with IBD [16, 17]. This is evident throughout the literature in the proportion of IBD patients experiencing depression and anxiety [16, 18]. In return, higher depression and anxiety in IBD patients have been linked to further exacerbation of symptoms [19], recurrent hospital admission, non-compliance and poor quality of life [20, 21]. Thus, addressing the disease management of IBD in a more holistic way that acknowledges the psychological burden of the disease, should be reflected in the IBD services.

### Models of integrated IBD care in other countries

Previous attempts to integrate IBD care in a holistic way have been seen in a number of countries as described by Mikocka-Walus [22]. For example in Adelaide (Australia) an IBD service model of care has been developed based on the recommendation of the British IBD Standards [9]. The model involves collaboration between all specialists involved in the care (gastroenterologists, IBD nurses and clinical/health psychologists as a core specialists and surgeons and dieticians as resources available), as well as number of services led by IBD nurses [22]. Similar patient focused models of IBD services based on multidisciplinary integration of activities is seen in Italy at Istituto Clinico Humanitas in Milan, Netherlands at the Erasmus Medical Centre and Canada at the IBD clinic at Winnipeg [22]. However, although the above examples use a holistic approach to care for IBD patients, those models are not a representative of common organisation and practice of services in the respective countries.

### Crohn’s and colitis UK

Crohn’s and Colitis UK is a charity organisation founded in 1979 dedicated to improve lives of people affected by Crohn’s disease and ulcerative colitis. The 28,000 members include not only patients, but their families, health professionals and others who support the work for improving quality of life and clinical care for people with Crohn’s disease, ulcerative colitis and other IBD diseases.

This paper reports the findings from the free text data of an online survey about the perceptions and experiences of service users of the current IBD services across Scotland. The study was part of a wider survey and the first of its kind in Scotland.

### Aim

To explore IBD patients’ perspectives and experiences of current IBD services and make recommendations for future service development.

## Method

The consolidated criteria for reporting qualitative research (COREQ) was used to guide the structure of this paper.

### Study design

This qualitative study was part of a wider national on-line survey on patient perspectives and experiences with current IBD services across the NHS in Scotland. The cross-sectional survey was made available between March and May 2014. Open invitations to take part in the survey were placed on the website of Crohn’s and Colitis UK, Crohn’s and Colitis UK Newsletter and an online survey link was distributed by Crohn’s and Colitis UK members using social networks such as Facebook and Twitter.

### Participants and setting

The survey was organised and administered by Crohn’s and Colitis UK in collaboration with the Scottish Government as the first step of a pilot project for co-designing IBD services in a bid to improve standards of care for IBD in Scotland. Participants in the study were patients across Scotland with a diagnosis of IBD. Participants recruited fulfilled both of the inclusion criteria: 1) to have a diagnosis of Crohn’s disease or ulcerative colitis and 2) to receive their treatment in Scotland (see Additional file 1 for full survey). Any respondent who did not meet the above criteria was excluded from the survey analysis. There is an understanding that online surveys do not require separate consent form, however the introduction paragraph accompanying the survey link as well as the introductory paragraph of the survey, conveyed the information about the purpose and anonymity of the study and consent was obtained by virtue of completion of the study. Research Governance at NRES (National Research Ethics Service) Committee for North of Scotland reviewed and exempted the study from a formal ethical review.

### Questionnaire

The on-line questionnaire was developed and designed by patients with IBD and members of Crohn’s and Colitis UK using Survey Monkey. Survey Monkey is a web-based, flexible and secure survey development tool [23]. The survey had 22 questions in total and took no more than 10 min to answer (see Additional file 1). However, only the findings from the three open ended questions, (see research questions) are presented in this paper. All open responses were reviewed in detail to identify common themes.

### Research questions

1. Please tell us two things which you think are good about your NHS care?
2. Please tell us two things which you think would improve your NHS care?
3. Anything else you would like to tell us about living with IBD?

### Analysis

Two researchers (MS, LM) independently coded the data to minimize subjectivity. All data were analysed using a thematic analysis framework approach [24]. This method is a rigorous approach consisting of 6 phases that provides structure for qualitative data to be organised, coded and themes to be identified. Both researcher read all data twice in order to become familiarized with it. After familiarization with data, generating initial codes and searching for themes among codes independently, researchers met to discuss their findings and extract the core themes. Then, researchers reviewed, defined and named the themes before producing the final report of key themes and sub themes (see Table 1). A report on the findings was presented to Crohn’s and Colitis UK members and discussed with them.

**Table 1:** Themes and subthemes

Themes	Subthemes
Quality of life	a. Impact on emotional/mental health b. Impact on physical health c. Impact on social health d. Impact on occupational health
IBD clinicians and better access	–
Clear IBD care pathway	a. Access to psychological and dietician services b. More advice and practical support on living with IBD c. Better education for GPs and A&E staff d. Availability of IBD care facilities

### Rigour

Qualitative studies are often judged on the basis of trustworthiness and rigour [25]. Rigour has been described as a means by which integrity and competence are demonstrated within a study [26]. These criteria were upheld throughout the analysis by being independently conducted by two researchers. Researcher met at three points (after the third, fifth and sixth step) to discuss findings and rectify any differences. Rigour with sampling was ensured through purposive selection using both intensity sampling (experts in the subject—people living with IBD) and maximum variation sampling (diverse sample) to make the data “information rich” [27].

## Results

### Demographics

Out of the 777 participants that responded to the survey, 10 (1.29 %) were excluded from the analysis as they did not meet the eligibility criteria of receiving treatment in Scotland. Table 2 includes the demographic data and clinical characteristics of participants in the sample (note that not all participants answered all demographic questions).

**Table 2:** Baseline demographics and clinical characteristics of participants

Baseline	
Answering the 1^st^ question (*n*,%)	610 (78.50)
Answering the 2^nd^question (*n*,%)	600 (77.22)
Answering the 3^rd^ question (*n*,%)	419 (53.92)
Age (years) (*n*, %)	
Under 16	37 (4.85)
16–65	671 (88.06)
Over 65	54 (7.09)
Sex	
F (*n*, %)	406 (66.56)
M (*n*, %)	204 (33.44)
Last flair up (*n*, %)	
Less than 6 months ago	367 (50.62)
6–12 months ago	124 (17.10)
1–2 years ago	107 (14.76)
2–4 years ago	70 (9.66)
More than 4 years ago	57 (7.86)
Length of diagnosis (*n*, %)	
Less than a year	17 (2.33)
1–5 years	265 (36.35)
5–10 years	181 (24.82)
10–20 years	142 (19.47)
Over 20 years	124 (17.00)

#### Key themes

Three major themes emerged at the final point of analysis: quality of life, access to IBD clinicians and the need for an explicit IBD care pathway. The themes occurred across all of the three questions. Further to the main themes, separate areas or sub themes unfolded (see Table 1).

#### Quality of life

**Fig. 1:**
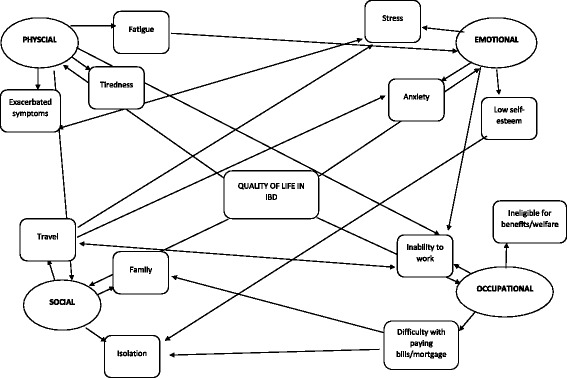
Thematic map showing interconnectedness between subthemes. The thematic map is showing how Quality of life is closely interconnected and affected by the physical, social, emotional and occupational health. In addition, it shows how for example physical symptoms (fatigue, tiredness & exacerbated symptoms) can have effect on specific aspects of social, occupational and emotional health and vice versa

Survey data suggested that some of the participants were concerned about the impact of IBD on their quality of life. Four categories or domains of quality of life were identified in the coding process. The categories that were consistently identified by the participants as being associated with quality of life, were: emotional/mental, physical, social and occupational health. The foundations for these sub themes are described separately alongside the verbatim quotes. Some of the quotes illustrate more than one sub theme simultaneously. See Fig. 1 for interconnectedness between the subthemes in this theme.

- Impact on emotional/mental health
  Participants described that living with IBD affects their emotional and mental health in a way that some consider it to be worse than a terminal illness (Table 3, quotes 1 & 2). Some participants described that the emotional impact for them is so great, that is affecting their confidence and sense of self (Table 3, quote 3). Others describe that the disease has left them feeling embarrassed and isolated with very little energy to form or keep up relationships (Table 3, quotes 4, 5, 6 &7).

**Table 3:** Quality of life: Impact on emotional/mental health

Quote no.	Quote
Quality of life: Impact on physical health	
1.	“The extreme tiredness is very hard to cope with.”(P315)
2.	“Feel tired even when asymptomatic. This affects my working ability and it would be very helpful to receive some sort of financial help like a tax credit or small benefit so I could always work 4 days a week without struggling.”(P412)
3.	“The fatigue that is debilitating and under estimated.” (P61)
4.	“My life has went on hold since diagnosis. Either because of pain or fatigue I have been unable to go on holiday, and worse still, my work has been hugely affected with long absences…”(P169)
Quality of life: Impact on social health	
5.	“Can be difficult when out for day and have to join queue for toilet-if use disable one can get dagger looks from other people who are me judging me as they don't realise what it is like as I do not look any different .” (P5)
6.	“I have lost 3 years of an important stage in my life to Crohn's and it has affected my friendships, my family and my education. “(P50)
7.	“Also feel quite fed up about having IBD because of travelling issues and being anxious about needing the toilet and having access to a toilet. The fear never goes away and you are always on the alert for where the nearest toilet is even if you hardly ever have to go urgently. “(P136)
Quality of life: Impact on occupational health	
8.	“It's very difficult and I worry a lot about not being able to work and pay my mortgage. Was refused benefit when I was off for around 6 months the last time.” (P41)
9.	“It isn't easy. Struggling more with working as I get older and coping with IBD.” (P102)
10.	“It is a constant struggle to get any welfare when not able to work. I have worked since I was 16 and paid my way but never there when I need it adding to stress levels” (P168)
  Participants described experiencing depression and anxiety that often followed exacerbation of symptoms, and therefore a identifying a need for acknowledgement and support for that aspect of the disease (Table 3, quotes 8, 9 & 10).
- Impact on physical health
  In addition to the mental health, participants identified the effects of constant tiredness, fatigue and pain (often present even in remission) on their physical health as a key area that needed to be addressed by practitioners. The quotes 1, 2, 3 & 4 (Table 4) demonstrate the participants’ experience of the physical effect the disease has on quality of life that they feel is underestimated.

**Table 4:** Quality of life: impact on physical, social and occupation health

Quote no.	Quote
Quality of life: Impact on emotional/mental health	
1.	“..at least cancer has the decency of killing you after torture. Not build you up and attack again…Also on antidepressants…” (P1)
2.	“it takes over your life….always have constant fear of what’s next.” (P148)
3.	“..impact on confidence is greatly affected..” (P8)
4.	“This illness is so debilitating and embarrassing…” (P131)
5.	“people don’t realise just how low you can get when having a flare up…it just drains you emotionally.” (P95)
6.	“its been horrendously isolating….has precluded me from contemplating seeking a partner.” (P139)
7.	“its horrible and upsetting and makes life a lot more difficult for the sufferer and sufferer’ family.” (P40)
8.	“More could be done on the counselling side of things too, depression and anxiety can follow a flare up.” (P36)
9.	“I find stress makes me flare up and feel that the nurses and GP's seem to be disregarding the help people need emotionally.” (P86)
10.	“Fatigue, stress and anxiety all make the condition worse and are brought about by it but no strategies are ever offered by NHS staff.” (P155)
- Impact on social health
  The quotes in this section describe the patients’ experiences of the impact that the disease has on every aspect of social and family life, leading to social isolation in some instances. Simple social activities that most people take for granted, such as going to town, or for a meal, can often be very stressful events due to not having access to disabled toilets (Table 4, quotes 5, 6 & 7). Participants wished for more support to be given to deal with these issues.
- Impact on occupational health
  The impact the condition has on occupation is particularly interlinked with the other domains of quality of life. Participants identified that coping with the disease and all the side effects, makes work very difficult for them (Table 4, quote 9). They also expressed the need for better help with benefits and welfare when unable to work (Table 4, quote 8). Some felt that the impact on occupational health presents a great cause for further distress (Table 4, quotes 9 & 10).

### IBD clinicians and better access

An important issue that participants highlighted was the need for more IBD nurses and gastroenterologists, together with improved access to them (Table 5, quote 3, 5, 8 & 9). Approximately one third of the participants agreed that having a named IBD nurse is one of the best things about their care (Table 5, quote 4, 6, 7). When asked about the part of the care that patients were happy about, participants described that nursing and consultant care were the parts of the service that they were happy about (Table 5, quote 2), but having better access to nurses and the gastroenterology team would improve the care for many (Table 5, quote 1 & 8).

**Table 5:** IBD clinicians and access

Quote no.	Quote
IBD Clinicians	
1.	“Quicker access to medical help over the weekend, my inflammatory flare ups always happen over the weekend. NHS 24 is hopeless over the weekend “(P78)
2.	“That they don't only support your physical disease but the emotional scars it leaves. That between the GI ward and IBD nurse it just feels like one big family. “(P78b)
3.	“More frequent gastroenterology visits with gastroenterologist or IBD nurse. Surely an IBD nurse for every sufferer in Scotland is achievable.”(P213)
4.	“IBD nurses are amazing people and having a named nurse to call when things go wrong is an amazing resource that is seriously undervalued.” (P58)
5.	I wasn't referred to the IBD nurse until I first went to hospital on the mainland. I live in a very remote area and knowing that this resource was available would have been very useful when I was first diagnosed. (P359)
6.	But having a nurse specialist is of great support and keeps things monitored much more closely (P89)
7.	Easier access to IBD and stoma nurse. (P64)
8.	More staff/nurses! The current ones are overworked and underpaid. (P85)

Some participants described that a particular improvement to services would be an access to an IBD nurse or consultant over the weekend. They highlighted that it is difficult to cope with exacerbated symptoms over the weekend if they are not able to access a knowledgeable healthcare professional (Table 5, quote 1).

### A more explicit IBD care pathway

Participants are looking for a more consistent and explicit care pathway that they can navigate with ease. For example, a participant expressed dissatisfaction with the care due to the poor coordination between departments which resulted in the patient staying at the bottom of their waitlists. In addition, the perception of the patient is that the different departments were working in isolation within their own deadlines, without working in a holistic person centered way (Table 6, quote 1).

**Table 6:** An explicit IBD care pathway

A more explicit IBD care pathway	
1.	“…but I kept being put back to day one in waiting lists in the next department, and felt there was a real lack of co-ordination between different departments in the hospital for outpatients, and was regularly made to feel that every department worked in isolation and only interested in their own deadlines…So I would improve the NHS by making different departments working together more rather than in tunnel vision. “(P145)
2.	“I have never seen the IBD nurse. I would like to know if he is available to me. Not sure who to ask.” (P234)
Access to psychological and dietician services	
3.	“I think more psychological help should be available, especially for people diagnosed as children, teenagers or young adults as it impacts your whole life and is still a taboo subject so it's difficult to talk about it” (P179)
4.	“A review by a dietician who is knowledgeable about IBD and allergic reactions of gut to chemicals”(P20)
Better information and practical support on living with IBD	
5.	“..more advice on how to live with IBD.”(P67)
6.	“Starter information pack giving new sufferers advice about lifestyle and diet.” (P170)
7.	“NHS care would also be improved by holistic approach that tackled non bowel symptoms such as fatigue.” (P213)
Better education for GPs and A&E staff	
8.	*“*There needs to be more belief in what patients are saying to health professionals about our symptoms and pain. I have felt on so many occasions that I'm not being believed. It minimises my pain and agony which affects my belief and trust in myself and my doctors too… It also makes me feel like I need to be on top of my game all the time, fighting and advocating for myself and the correct treatment. Sometimes I feel like its 'them vs me' when we should all be working for the same end goal at getting me better. This can be even more exhausting on top of the disease itself.” (P135)
Availability and coordination of IBD care facilities and services	
9.	“When in hospital I think putting u in a ward with a shared toilet is a bit hard.”(P184)
10.	“I have had very little follow up care after being diagnosed in 2012. My referral has been lost twice and I am still not under the care of a gastroenterologist or on any medication despite my diagnosis! (P768)

Other participants felt are left not knowing who to contact for advice when sick and have a flair up, without getting conflicting advice (Table 6, quote 2); or feel that are getting ‘lost’ in the system (Table 6, quote 3). According to the participants’ answers and suggestions towards service improvements in the IBD pathway, four main areas were identified:

- Access to psychological and dietician services:
  Participants repeatedly described the need for availability and access to psychological and dietician services as part of the IBD care pathway (Table 6, quotes 4 & 5 and Table 3, quote 8). This would enable many diagnosed with IBD to cope better with a condition and symptoms that are a taboo.
- Better information and practical support on living with IBD:
  This sub theme identifies the need for a more holistic approach to the condition, where all symptoms, including the extra-gastrointestinal ones are addressed early on. Quotes 6, 7 & 8 (Table 6) describe the need for advice and support being available to patients with IBD as part of the care package.
- Better education for GPs and A&E staff:
  Patients with IBD felt that health professionals have to be better educated about the condition. Participates often felt they had to ‘fight’ with clinical staff because of their lack of knowledge on IBD and often felt like they are on opposite ‘teams’. In addition, they recognised that it is very difficult or impossible to be an advocate for the disease and their care particularly at critical times, when their symptoms are exacerbated. Quote 9 in Table 6 comprehensively captures what other participants also described.
- Availability and coordination of IBD care facilities and services:
  Another area highlighted by patients was the availability of their own toilet on the ward, not having to share a room with others and the poor quality of hygiene in hospitals (Table 6, quote 10). In addition: quicker appointments, quicker diagnosis, regular follow up and at least, an annual review of care and local IBD facilities for patients living in the remote areas was among the other key areas identified by the participants in need of improvement (Table 6, quote 11).

## Discussion

The primary aim of this survey was to explore IBD patients’ experiences of current services and make recommendations for future service development. The strength of this study lies in: the key aim to co-design IBD services in the NHS in Scotland; the diverse sample of patients (making it a cross sectional representation of patients with IBD in comparison to other similar studies [28–30]) and the systematic method of data analysis. To the authors’ knowledge, this is the first study to qualitatively explore the IBD patients’ experiences and perspectives about their NHS IBD care in Scotland using a cross-sectional analysis. The findings have given a clear insight into what is important to patients with IBD.

Participants expressed that while living with IBD, their needs are wider than just being medical or biological. Coping with the extra-gastrointestinal manifestations, side effects from medications and particularly the distress from the relapsing nature of the condition was something that participants found challenging when not supported adequately. They also acknowledged that exacerbation of symptoms often caused extra stress associated with travel to and from work. This in addition to the tiredness, fatigue and pain, made working full time very difficult. The participants also reported that the disabling impact of IBD was not always recognised by the employers and the public in general and therefore suggested that strategies needed to be put in place to raise awareness on the disabling nature of IBD.

The data reflects that the participants perceived that the disease had wider effect on their quality of life (inclusive of mental/emotional, physical, social and occupational health). This highlighted the need for a more holistic approach to their care, which includes psychological, counselling and dietician services at least at the point of diagnosis. This is supported by evidence from the literature that emotional and dietary support plays a key part in the quality of life for patients with IBD and should be met accordingly [31–35].

In addition, they suggested practical advice and strategies on how to live with IBD as soon as diagnosed. For example, one of the main symptoms, poor bowel control, is a major concern for patients with IBD, because it limits their personal, working and social lives in complex ways; thus practical advice at diagnosis would be invaluable for better quality of life [36]. This is also in line with evidence from recent literature that highlights the limitation of a purely GI-centric view of IBD [37], advocating the need for a biopsychosocial model in gastroenterology particularly for chronic GI conditions [38].

Participants were most satisfied with their care when had good access to IBD clinicians and IBD patients with a named IBD nurse reported higher satisfaction. In contrast, some service users were not aware of such a specialist role, and many participants described that improved access to an IBD clinician (IBD nurse and gastroenterologist) would be an advantage when having exacerbated symptoms. In fact, previous studies suggest that hospital visits were reduced and remission increased among patients with IBD when IBD specialist nurse was involved in their care management [39, 40].

And finally, participants described that one of the difficulties with their care was not having a clear IBD care pathway. They felt that there were gaps in communication across different departments and as a result, their care was not well coordinated which resulted in waiting for appointments with specialists for months or have even been referred to the wrong place. The relapsing nature of the disease can have a restrictive effect on many aspects of daily life for patients. Thus, the services for these patients should not only be comprehensive but also easily accessible and well-coordinated. This requires integration and coordination of different health care sectors, medical and non-medical professionals, social and health care facilities and funding agencies [41]. In return, integrated care with a clear pathway for patients could play a significant role in determining health-related quality of life for IBD patients [22, 42].

Thus, findings that illustrate the patients’ perspective about the care received, are worth considering by clinicians, researchers and policy makers aiming at improving the standards of care for patients with IBD. This study has provided strong evidence from patients’ perspectives that a purely GI-centric view of IBD is no longer an adequate way of addressing the disease concerns and attention to mental and social health should not be overlooked by healthcare providers who often tend to focus only on gastrointestinal symptoms [16]. Thus, the patients’ accounts from the study make a stronger case for adopting the biopsychosocial model in IBD care. Although the biopsychosocial model is not new in gastroenterology and IBD care and has been applied in various degrees in different countries as mentioned before, nonetheless is still rare even more so on a national scale.

This model identifies the need for specialists from different disciplines to work together in a holistic and coordinated way. This means that the various specialists (services/teams/clinicians) communicate regularly, have a collective referral system and work together to offer integrated treatment to patients. In return, this would lessen the confusion not only among patients, but also among healthcare providers when giving advice or making referrals. This in addition, could prevent patients being ‘lost’ in the system and allow an early engagement with services when needed before escalating to emergency admissions. Patients also require easy treatment access to IBD specialists such as IBD nurses. Patients expressed a greater satisfaction with their care when they consulted IBD nurses. Evidence also suggests that having IBD nurses with extended roles results in improvement in health outcomes and saving in healthcare costs [22].

### Limitations

A limitation of this study was that all study information had to be given on the first ‘page’ of the survey and it was not possible to provide an oral explanation or to take oral consent. However, as with paper-based information sheet, the ‘first page’ of the survey identified who the researcher was, reason for conducting the survey, what the survey data will be used for and anonymity of the survey [43].

Another limitation is that the experiences of the respondents may not be representative of all IBD service users in Scotland as not everyone with IBD responded to the survey. Patients with IBD that do not have access to the media that was used to distribute the survey were not able to participate. However, in qualitative studies, the strength lies with the richness of data, which was received from those that participated, providing invaluable feedback about the current IBD services.

## Conclusions

In conclusion, this study described the personal experiences of IBD services by the users who identified key areas for improvement. Although these findings do not represent the views of all patients with IBD in Scotland, the findings give a clear insight into some practical recommendations for treatment providers, service managers and policy makers to enhance the IBD standard of care. These findings also provide information for service planners and policy makers on the importance and value of co design both for designing and restructuring of services that are relevant for service users.

## Abbreviations

A&E-Accident and Emergency; IBD-Inflammatory Bowel Disease; NHS-National Health Service; NRES-National Research Ethics Service; UK-United Kingdom

## Additional files

Additional file 1: Full Scotland IBD questionnaire. Description of data: Supplementary Material 1: This is the full IBD survey questionnaire that was used across Scotland to collect the qualitative (reported in this manuscript) and quantitative data (not reported in this manuscript). (PDF 188 kb)

## References

[1] Binder T, Brandt E, Gregory J (2008). Design participation (−s)—a creative commons for ongoing change. CoDesign: Int J CoCreation Des Arts.

[2] Alam I (2002). An exploratory investigation of user involvement in new service development. J Acad Mark Sci.

[3] Edvardsson B, Gustafsson A, Kristensson P, Witell L, Maglio PP, Kieliszewski CA, Spohrer JC (2010). Service innovation service innovation and customer Co-development. Handbook of service science.

[4] Kujala S (2003). User involvement: a review of the benefits and challenges. Behav Inform Technol.

[5] Sanders EB, Stappers PJ (2008). Co-creation and the new landscapes of design. Co-des.

[6] Pascoe GC (1983). Patient satisfaction in primary health care: a literature review and analysis. Eval Program Plann.

[7] Goldstein E, Cleary PD, Langwell KM, Zaslavsky AM, Heller A (2001). Medicare managed care CAHPS®: a tool for performance improvement. Health Care Financ Rev.

[8] Landon BE, Zaslavsky AM, Bernard SL, Cioffi MJ, Cleary PD (2004). Comparison of performance of traditional Medicare vs Medicare managed care. JAMA.

[9] IBD Standards Working Group: Quality Care: Service Standards for the Healthcare of People who have Inflammatory Bowel Disease (IBD). 2009. http://www.bsg.org.uk/attachments/160_IBDstandards.pdf.

[10] IBD Standards Working Group: IBD Standards: Standards for the Healthcare of People who have Inflammatory Bowel Disease (IBD). 2013. http://www.ibdstandards.org.uk/uploaded_files/ibdstandards.pdf.

[11] Lakatos PL. Recent trends in the epidemiology of inflammatory bowel diseases: up or down? World J Gastroenterol. 2006;12(38):6102–08. 10.3748/wjg.v12.i38.6102 PMC4088101 17036379

[12] Stone MA, Mayberry JF, Baker R (2003). Prevalence and management of inflammatory bowel disease: a cross-sectional study from central England. Eur J Gastroenterol Hepatol.

[13] Sawczenko A, Sandhu B, Logan R, Jenkins H, Taylor C, Mian S, Lynn R. Prospective survey of childhood inflammatory bowel disease in the British isles. Lancet. 2001;357(9262):1093–4. 10.1016/s0140-6736(00)04309-9 11297962

[14] Henderson P, Wilson DC (2012). The rising incidence of paediatric-onset inflammatory bowel disease. Arch Dis Child.

[15] Andrews J, Mountifield R, Van Langenberg D, Bampton P, Holtmann G (2010). Un-promoted issues in inflammatory bowel disease: opportunities to optimize care. Intern Med J.

[16] Graff LA, Walker JR, Bernstein CN (2009). Depression and anxiety in inflammatory bowel disease: a review of comorbidity and management. Inflamm Bowel Dis.

[17] Zhang CK, Hewett J, Hemming J, Grant T, Zhao H, Abraham C, Oikonomou I, Kanakia M, Cho JH, Proctor DD. The influence of depression on quality of life in patients with inflammatory bowel disease. Inflamm Bowel Dis. 2013;19(8):1732–9. 10.1097/MIB.0b013e318281f395 PMC4623582 23669400

[18] Mikocka-Walus A, Knowles SR, Keefer L, Graff L (2016). Controversies revisited: a systematic review of the comorbidity of depression and anxiety with inflammatory bowel diseases. Inflamm Bowel Dis.

[19] Mittermaier C, Dejaco C, Waldhoer T, Oefferlbauer-Ernst A, Miehsler W, Beier M, Tillinger W, Gangl A, Moser G. Impact of depressive mood on relapse in patients with inflammatory bowel disease: a prospective 18-month follow-up study. Psychosom Med. 2004;66(1):79–84. 10.1097/01.psy.0000106907.24881.f2 14747641

[20] Bernal I, Domenech E, Garcia-Planella E, Marín L, Mañosa M, Navarro M, Cabré E, Gassull MA. Medication-taking behavior in a cohort of patients with inflammatory bowel disease. Dig Dis Sci. 2006;51(12):2165–9. 10.1007/s10620-006-9444-2 17086434

[21] Faust AH, Halpern LF, Danoff-Burg S, Cross RK (2012). Psychosocial factors contributing to inflammatory bowel disease activity and health-related quality of life. Gastroenterol hepatol.

[22] Mikocka‐Walus AA, Andrews JM, Bernstein CN, Graff LA, Walker JR, Spinelli A, Danese S, van der Woude, C Janneke, Goodhand J, Rampton D. Integrated models of care in managing inflammatory bowel disease: a discussion. Inflamm Bowel Dis. 2012;18(8):1582–7. 10.1002/ibd.22877 22241699

[23] Gordon A (2002). SurveyMonkey. com—Web-based survey and evaluation system: http://www. SurveyMonkey. com. Internet High Educ.

[24] Braun V, Clarke V (2006). Using thematic analysis in psychology. Qual res psychol.

[25] Creswell JW. Research design: Qualitative, quantitative, and mixed methods approaches. London: Sage publications; 2013.

[26] Tobin GA, Begley CM (2004). Methodological rigour within a qualitative framework. J Adv Nurs.

[27] Patton MQ. Qualitative research. John Wiley & Sons, Ltd; 2005. doi:10.1002/0470013192.bsa514.

[28] Pomeroy D (2013). The infammatory bowel disease (IBD) service: patient satisfaction survey. Gastrointest Nurs.

[29] Sadlo A, Altevers J, Peplies J, Kaltz B, Classen M, Bauer A, Koletzko S, Timmer A. Measuring satisfaction with health care in young persons with inflammatory bowel disease—an instrument development and validation study. BMC Health Serv Res. 2014;14:97–6963. 10.1186/1472-6963-14-97 PMC3946022 24581043

[30] Vasudevan A, Arachchi A, van Langenberg DR (2013). Assessing patient satisfaction in inflammatory bowel disease using the QUOTE-IBD survey: a small step for clinicians, a potentially large step for improving quality of care. J Crohn’s Colitis.

[31] Smith GD, Watson R, Roger D, McRorie E, Hurst N, Luman W, Palmer KR. Impact of a nurse-led counselling service on quality of life in patients with inflammatory bowel disease. J Adv Nurs. 2002;38(2):152–60. 10.1046/j.1365-2648.2002.02159.x 11940128

[32] Prince A, Whelan K, Moosa A, Lomer MC, Reidlinger DP (2011). Nutritional problems in inflammatory bowel disease: the patient perspective. J Crohn’s Colitis.

[33] Miehsler W, Weichselberger M, Öfferlbauer‐Ernst A, Dejaco C, Reinisch W, Vogelsang H, Machold K, Stamm T, Gangl A, Moser G. Which patients with IBD need psychological interventions? a controlled study. Inflamm Bowel Dis. 2008;14(9):1273–80. 10.1002/ibd.20462 18393373

[34] Ghosh S, Mitchell R (2007). Impact of inflammatory bowel disease on quality of life: results of the European federation of Crohn’s and ulcerative colitis associations (EFCCA) patient survey. J Crohn’s Colitis.

[35] McCombie AM, Mulder RT, Gearry RB (2013). Psychotherapy for inflammatory bowel disease: a review and update. J Crohn’s Colitis.

[36] Dibley L, Norton C (2013). Experiences of fecal incontinence in people with inflammatory bowel disease: self-reported experiences among a community sample. Inflamm Bowel Dis.

[37] Moradkhani A, Beckman LJ, Tabibian JH (2013). Health-related quality of life in inflammatory bowel disease: psychosocial, clinical, socioeconomic, and demographic predictors. J Crohns Colitis.

[38] Knowles SR, Mikocka-Walus AA. Psychological Aspects of Inflammatory Bowel Disease: A Biopsychosocial Approach. London: Routledge; 2014.

[39] Nightingale AJ, Middleton W, Middleton SJ, Hunter JO (2000). Evaluation of the effectiveness of a specialist nurse in the management of inflammatory bowel disease (IBD). Eur J Gastroenterol Hepatol.

[40] Hernandez-Sampelayo P, Seoane M, Oltra L, Marin L, Torrejon A, Vera MI, Garcia V, Lazaro P, Parody E, Blasco AJ, Casellas F. Contribution of nurses to the quality of care in management of inflammatory bowel disease: a synthesis of the evidence. J Crohns Colitis. 2010;4(6):611–22. 10.1016/j.crohns.2010.08.009 21122570

[41] Raspe H, Conrad S, Muche-Borowski C (2009). Evidence-based and consented pathways for patients with inflammatory bowel diseases (IBD). Z Gastroenterol.

[42] van der Eijk I, Vlachonikolis IG, Munkholm P, Nijman J, Bernklev T, Politi P, Odes S, Tsianos EV, Stockbrügger RW, Russel MG. The role of quality of care in health‐related quality of life in patients with IBD. Inflamm Bowel Dis. 2004;10(4):392–8. 10.1097/00054725-200407000-00010 15475747

[43] Kraut R, Olson J, Banaji M, Bruckman A, Cohen J, Couper M (2004). Psychological research online: report of board of scientific Affairs’ advisory group on the conduct of research on the internet. Am Psychol.

