# Supplementary material for: Co-designing inflammatory bowel disease (Ibd) services in Scotland: findings from a nationwide survey
Source: BMC Health Serv Res. 2016 Jul 8;16:231. doi: 10.1186/s12913-016-1490-7 (PMC4939055; doi:10.1186/s12913-016-1490-7)
Supplement: Supplementary file 1 — Full Scotland IBD questionnaire. Description of data: Supplementary Material 1: This is the full IBD survey questionnaire that was used across Scotland to collect the qualitative (reported in this manuscript) and quantitative data (not reported in this manuscript). (PDF 188 kb) [file 12913_2016_1490_MOESM1_ESM.pdf]

Scotland IBD Steering Group Survey

Introduction

Crohn's and Colitis UK is seeking the views of people with IBD in Scotland, to better understand the standards of treatment and care that they are receiving. This includes diagnosis, symptoms and the length of time you wait for advice and treatment.

We will use the trends identified, to demonstrate the need for further improvements in standards of care for everyone with these conditions in Scotland, at a meeting of influential MSPs and patients later this year.

This is a short, anonymous survey that will take up to 10 minutes to complete.

*1. Do you receive your treatment in Scotland?

- ◯ Yes
- ◯ No

2. Are you

- ◯ Male
- ◯ Female

3. Are you...

- ◯ Under 16
- ◯ 16 - 65
- ◯ Over 65
- ◯ Filling in this on behalf of a child

4. What was the year of your diagnosis of Crohn's Disease or Ulcerative Colitis

5. Do you have an IBD Nurse?

- ☐ Yes
- ☐ No

6. Do you have an annual review for your Crohn's Disease/Ulcerative Colitis

- ○ Yes
- ○ No

7. Who by?

- ○ Gastroenterologist
- ○ IBD Nurse
- ○ GP
- ○ Other

Other (please specify)

[ ]

8. How long ago were you last poorly (flare up) with Crohn’s Disease or Ulcerative Colitis?

- ○ Less than 6 months DEL
- ○ 6 - 12 months ago DEL
- ○ 1 - 2 years ago DEL
- ○ 2 - 4 years ago DEL
- ○ More than four years

If you were last poorly more than four years ago, please specify below...

[ ]

9. What were the symptoms of your most recent flare up?

- ☐ Diarrhoea
- ☐ Change in bowel habit DEL
- ☐ Urgency
- ☐ Incontinence DEL
- ☐ Rectal bleeding DEL
- ☐ Active fistula/abscess DEL
- ☐ Abdominal pain DEL
- ☐ Constipation
- ☐ Vomiting DEL
- ☐ Weight loss
- ☐ Loss of appetite DEL
- ☐ Symptoms outside the bowel (skin, eye, joint inflammation)

Other (please specify)

10. What did you do?

- ○ Contact health professional
- ○ Ignore
- ○ Self-treat

Other (please specify)

11. Did you follow guidelines agreed with a Health professional for your self treatment

- ○ Yes
- ○ No

Please explain

12. Who did you contact?

- ◯ A & E DEL
- ◯ IBD Nurse DEL
- ◯ Gastroenterologist
- ◯ GI Ward DEL
- ◯ GP DEL
- ◯ Secretary/Admin staff

Other (please specify)

13. How long did you have to wait for advice, after contacting your health service...

- ◯ Less than 24 hours DEL
- ◯ 24 – 48 hours DEL
- ◯ 2 - 5 days DEL

More (please specify)

14. What action was taken

- [ ] No action taken DEL
- [ ] Advice/information about managing my illness DEL
- [ ] Old medication altered DEL
- [ ] New medication begun DEL
- [ ] Nutritional therapy begun DEL
- [ ] Blood test
- [ ] Stool test DEL
- [ ] Endoscopy DEL
- [ ] Faecal Calprotectin test DEL
- [ ] X-ray DEL
- [ ] Scan
- [ ] Referral to a Gastroenterologist DEL
- [ ] Referral to a Surgeon DEL
- [ ] Referral to a Rheumatologist DEL
- [ ] Referral to a Dermatologist DEL
- [ ] Referral to an IBD nurse DEL
- [ ] Referral to a Dietitian DEL
- [ ] Referral to a Counsellor/Psychologist
- [ ] Admission to hospital DEL

Other (please specify)

____

15. Would you have liked to have been referred to

- [ ] Gastroenterologist DEL
- [ ] Surgeon DEL
- [ ] Rheumatologist DEL
- [ ] Dermatologist DEL
- [ ] IBD nurse DEL
- [ ] Dietitian DEL
- [ ] Counsellor/Psychologist DEL

Other (please specify)

____

16. Would you have liked a referral to any of the following

- [ ] Occupational Therapist DEL
- [ ] Careers Adviser DEL
- [ ] Social Worker DEL
- [ ] Vocational Rehabilitation Service

Other (please specify)

17. Overall how satisfied were you with your treatment

Very satisfied	Quite satisfied	Not satisfied	Very unsatisfied
○	○	○	○

18. During this flare up did you take time off:-

- ○ Yes
- ○ No
- ○ Not Applicable

19. How long?

- ○ Less than one week DEL
- ○ 1 - 2 weeks DEL
- ○ 2 – 4 weeks
- ○ MoreDEL

Please specify

20. Please tell us two things which you think are good about your NHS care:-

21. Please tell us two things which you think would improve your NHS care:-

22. Anything else you would like to tell us about living with IBD?
